# *AAV9‐SORL1 gene therapy* for Alzheimer’s disease

**DOI:** 10.1002/alz.088971

**Published:** 2025-01-09

**Authors:** Olav M. Andersen, Yasir H. Qureshi, Anne Mette Gissel Jensen, Andrew Williams, Jessica E. Young, Mimi Roy, Joel Klein, Rob Armstrong, Kalpana M. Merchant

**Affiliations:** ^1^ Retromer Therapeutics, New York, NY USA; ^2^ Aarhus University, Aarhus C Denmark; ^3^ University of Washington, Seattle, WA USA; ^4^ Institute for Stem Cell and Regenerative Medicine, Seattle, WA USA

## Abstract

**Background:**

Convergent evidence indicates that deficits in the endosomal recycling pathway underlies pathogenesis of Alzheimer’s disease (AD). *SORL1* encodes the retromer‐associated receptor SORLA that plays an essential role in recycling of AD‐associated cargos such as the amyloid precursor protein and the glutamatergic AMPA receptor. Importantly, loss of function pathogenic *SORL1* variants are associated with AD. Moreover, both SORLA and retromer protein levels are reduced in the most vulnerable regions of AD brains. Thus, restoration of SORLA levels and function *via* viral transduction is an attractive therapeutic strategy. Unfortunately, the full‐length *SORL1* is too large to be packaged into a viral capsid for gene therapy.

**Method:**

We identified and tested endosomal recycling effects of a truncated *SORL1* mini‐gene construct after transient transfections in HEK293 cells. We developed a gene therapy lead by packaging *SORL1* mini‐gene into AAV9. This lead was tested for expression and activity in neurons differentiated from human induced pluripotent cells (hiPSC) as well as *in vivo* after brain infusions in wild‐type mice and *SORL1* haploinsufficient Göttingen minipigs by biochemistry and immunohistochemistry.

**Result:**

Expression of the *SORL1* mini‐gene in HEK293 cells increased endosomal recycling of full‐length SORLA as shown by elevated cell surface expression and shedding of the SORLA mini receptor. In hiPSC neurons, AAV9‐*SORL1* treatment resulted in strong endosomal expression of the SORLA mini‐receptor that co‐localized with components of the retromer complex in endosomes. *In vivo*, we observed dose‐dependent transduction and expression of SORLA mini‐receptor in neurons, which localized to endosome‐like punctate structures. Evaluation of functional effects is underway.

**Conclusion:**

We conclude that the AAV9‐*SORL1* mini‐gene expresses SORLA mini‐receptor in neurons which resides in intracellular vesicular endosomal structures together with the retromer complex and has the capacity to boost the endosomal recycling pathway. Thus, the *SORL1* mini‐gene is a promising candidate for gene‐therapy for AD patients.

